# Rigid respiration: fulminant pulmonary fibrosis after COVID-19

**DOI:** 10.1016/j.ebiom.2022.104428

**Published:** 2022-12-27

**Authors:** David K. Meyerholz

**Affiliations:** Department of Pathology, University of Iowa Carver College of Medicine, Iowa City, Iowa, 52242, USA

Coronavirus disease of 2019 (COVID-19) is an infection caused by severe acute respiratory syndrome-coronavirus-2 (SARS-CoV-2), a novel, zoonotic coronavirus. COVID-19 cases in humans were first recognized near Wuhan China in late 2019, but the infection rapidly spread around the globe and was declared a pandemic by the World Health Organization only a few months later in 2020.^1^^,^^2^ Most individuals infected by SARS-CoV-2 have a mild clinical course with common cold-like respiratory tract symptoms and clinical involvement in other organ systems is not uncommon. For individuals with severe COVID-19 infection, lung disease is a common pathological feature that can progress to acute respiratory distress syndrome (ARDS) and this is associated with hospitalization, intensive care unit admission, mechanical ventilation and even mortality.^2^^,^^3^

Acute clinical manifestations of COVID-19 are generally well-defined, but the emergence of post-acute sequelae in survivors following primary infection and sequential reinfection are increasingly documented and recognized.^4^^,^^5^ Within the spectrum of post-acute sequelae, pulmonary fibrosis is a major complication where diminished lung function can require lung transplantation or be a contributing factor to mortality.^5^ Pulmonary fibrosis occurs when aberrant collagens are deposited in the extracellular matrix and this results in a rigid lung that lacks the compliance ("stretchability") needed for routine respiration. Reports of post-COVID-19 pulmonary fibrosis (PCPF) are more associated with clinical factors consistent with severe disease including: higher lung CT scores, ICU admission, mechanical ventilation and length of hospitalization.^6^ The pathogenesis of this emergent syndrome urgently needs to be studied as cellular and molecular pathways contributing to PCPF are thought to be multifactorial and poorly-defined.^6^

Affected tissues like the lung can be directly studied to better correlate clinical observations, clarify disease pathogenesis, and define cellular and molecular mechanisms of COVID-19.^1^ In the December 2022 issue of *eBioMedicine*, Jyothula and colleagues studied lung tissues collected from a cohort of COVID-19 patients at the time of lung transplant, an average of approximately four months following COVID-19 diagnosis.^7^ Deteriorated and nonresolvable lung function consistent with ARDS made their transplants a necessity.^7^ Histopathological examination of lungs from the COVID-19 cohort showed acute to chronic stages (i.e., effusive, organizing and fibrotic stages) of diffuse alveolar disease (DAD), consistent with ARDS. The COVID-19 lung cohort were compared against control lungs from tissue-donors and explanted lung tissue from idiopathic pulmonary fibrosis (IPF) patients. Molecular analysis showed that COVID-19 lungs had a clustered and increased expression in extracellular matrix pathways relative to control lungs. As one example, collagen triple helix repeat containing 1 (CTHRC1+), a recently described marker for profibrotic fibroblasts,^8^ had elevated expression localized to fibroblasts admixed with PCPF lesions. Cumulatively, this study suggests a hyper-fibrotic phenotype that is distinct from IPF lungs and it may contribute to the fulminant pulmonary fibrosis seen in PCPF.

The study by Jyothula and colleagues illuminates clinical features and remodeling pathways linked with advanced stages of PCPF lung disease,^7^ but several questions remain unanswered. First, the Jyothula et al. study focused on end stage lung disease requiring transplant, but what are the prior cellular and molecular pathways that initiate or accelerate this process into fulminant PCPF and how can we monitor these transition stages? Some have proposed that consistent use of defined criteria in CT-based diagnosis of PCPF can help elucidate the kinetics of PSPF development and prevent over-estimations in its incidence.^9^ Next, persistent inflammation is thought to be a pro-fibrotic factor, so might an aberrant inflammatory response to SARS-CoV-2 infection initiate or accelerate progression to PCPF? A recent study found that as far as one year after COVID-19 infection, some individuals retained an interstitial pattern of lung disease with elevated pro-neutrophilic inflammatory markers in the peripheral blood.^10^ Further, monocytes exposed to SARS-CoV-2 virus, but not influenza or viral RNA analogs, induced a profibrotic signature similar to CD163+ macrophages that accumulate in COVID-19 lung.^5^ 3) Finally, can targeted therapies prevent or resolve PCPF lesions with meaningful outcomes? Clinical trials are being evaluated in COVID-19 patients to test whether anti-inflammatory and antifibrotic therapies show efficacy in mitigating lung fibrosis.^9^ Whether these therapies will make clinically significant impacts on respiratory function or quality of life for people following COVID-19 infection remains unknown.^9^

## Contributors

DKM wrote the manuscript, revised the final manuscript, and is responsible for summarizing all the data.

## Declaration of interests

Author declares no competing interests. DKM acknowledge the support of HL163556, HL152960, DK054759, HL091842, HL147366 and 10.13039/100000897Cystic Fibrosis Foundation.
